# Circadian Clock and OxInflammation: Functional Crosstalk in Cutaneous Homeostasis

**DOI:** 10.1155/2020/2309437

**Published:** 2020-04-23

**Authors:** Elena Frigato, Mascia Benedusi, Anna Guiotto, Cristiano Bertolucci, Giuseppe Valacchi

**Affiliations:** ^1^Department of Life Sciences and Biotechnology, University of Ferrara, I-44121 Ferrara, Italy; ^2^Department of Medical and Surgical Sciences, University of Ferrara, I-44121 Ferrara, Italy; ^3^NC State University, Plants for Human Health Institute, Department of Animal Sciences, NC Research Campus, Kannapolis, NC 28081, USA

## Abstract

Circadian rhythms are biological oscillations that occur with an approximately 24 h period and optimize cellular homeostasis and responses to environmental stimuli. A growing collection of data suggests that chronic circadian disruption caused by novel lifestyle risk factors such as shift work, travel across time zones, or irregular sleep-wake cycles has long-term consequences for human health. Among the multiplicity of physiological systems hypothesized to have a role in the onset of pathologies in case of circadian disruption, there are redox-sensitive defensive pathways and inflammatory machinery. Due to its location and barrier physiological role, the skin is a prototypical tissue to study the influence of environmental insults induced OxInflammation disturbance and circadian system alteration. To better investigate the link among outdoor stressors, OxInflammation, and circadian system, we tested the differential responses of keratinocytes clock synchronized or desynchronized, in an *in vitro* inflammatory model exposed to O_3_. Being both NRF2 and NF-*κ*B two key redox-sensitive transcription factors involved in cellular redox homeostasis and inflammation, we analyzed their activation and expression in challenged keratinocytes by O_3_. Our results suggest that a synchronized circadian clock not only facilitates the protective role of NRF2 in terms of a faster and more efficient defensive response against environmental insults but also moderates the cellular damage resulting from a condition of chronic inflammation. Our results bring new insights on the role of circadian clock in regulating the redox-inflammatory crosstalk influenced by O_3_ and possibly can be extrapolated to other pollutants able to affect the oxinflammatory cellular processes.

## 1. Introduction

Circadian rhythms are biological oscillations that occur with an approximately 24 h period and are synchronized to cyclic environmental cues, such as light-dark cycles and timing of food intake [1]. In mammals, the circadian system encompasses a central circadian pacemaker in the suprachiasmatic nucleus (SCN) of the hypothalamus which coordinates autonomous peripheral oscillators, localized in almost all tissues and organs, including the liver, heart, skeletal muscle, and skin [2–5]. Functional clock in each of these tissues optimizes cellular homeostasis and responses to environmental stimuli [6]. At the cellular level, the clock is defined as a transcriptional and translational feedback loop oscillator. The core loop consists of the CLOCK:BMAL1 heterodimer, which activates the transcription of period (PER) and cryptochrome (CRY). PER and CRY proteins form heterodimeric complexes that inhibit their own transcription by suppressing the activity of CLOCK:BMAL1 [7]. The molecular clock drives intrinsic daily rhythms of physiology and behavior. The coordinated activity of central and peripheral oscillators can be referred to as the circadian timing system. A growing collection of data suggests that chronic circadian disruption caused by novel lifestyle risk factors such as shift work, travel across time zones, or irregular sleep-wake cycles has long-term consequences for human health [8, 9], causing cellular dysfunction and chronic diseases. Among the multiplicity of physiological systems hypothesized to have a role in the onset of pathologies in case of circadian disruption, there are redox-sensitive defensive pathways and inflammatory machinery [10, 11]. The effects of dysregulated circadian system on cellular defensive enzyme activities were previously reported, and recent investigations have further elucidated the molecular mechanisms that are possibly the link between the circadian clock and the maintenance of cellular redox homeostasis [12]. Moreover, a growing collection of data focus on the deep interconnection between altered redox homeostasis and inflammatory pathways (OxInflammation), since it seems that the imbalance of those two critical pathways can contribute to initiation, development, and progression of several disorders [13–16].

Due to its location and barrier physiological role, the skin is a prototypical tissue to study the influence of environmental insults induced OxInflammation disturbance and circadian system alteration. There are a lot of data suggesting the correlation between chronic and relapsing cutaneous inflammatory diseases, such as psoriasis and atopic dermatitis, and aberrant circadian system [17–19]. Moreover, a growing set of data [2, 20–23] suggests a clear correlation between the cellular circadian system and oxidative stress. Concerning the skin, our group has previously demonstrated that in human keratinocytes, which comprise ~95% of the cells within the epidermis, endogenous circadian clock is involved in the cellular response to oxidative stress. In particular, after the exposure to ozone (O_3_), one of the most toxic outdoor pollutants, the clock-synchronized keratinocytes exhibit a more efficient antioxidant response compared to arrhythmic ones, attested by a quicker activation of the master defensive cellular transcription factor, nuclear factor erythroid 2-related factor 2 (NRF2) [12].

Starting from these evidences, to further investigate the link between circadian system, redox homeostasis, and inflammation, we envisage a scenario in which we tested the differential response of clock-synchronized or clock-desynchronized keratinocytes, in human keratinocytes exposed to O_3_ insult in the presence of LPS.

It is well demonstrated that occurrence of oxidative stress contributes to generate an induction of proinflammatory status [24–26], so we hypothesized that circadian deregulation could compromise the crosstalk between these two pathways.

Since NRF2 and NF-*κ*B are the two key transcription factors that regulate cellular responses to oxidative stress and inflammation, we further analyzed the crosstalk between the two transcription factors in the presence of different challenges such as O_3_ and LPS.

A better understanding of the interactions between the circadian system and the cell physiological pathways is required to better apprehend the role of the clock in pathological development.

## 2. Materials and Methods

### 2.1. Cell Culture

HaCaT cell line (obtained from American Type Culture Collection, ATCC) was cultured in DMEM (Lonza, Milan, Italy) supplemented with 10% fetal bovine serum (FBS, EuroClone, Milan, Italy), 1% of L-glutamine (Lonza, Milan, Italy), and 1% of penicillin/streptomycin antibiotics (Lonza, Milan, Italy) at 37°C in 5% CO_2_. To induce chronic inflammation, cells were seeded into 6 well-plates, cultured to 60-70% confluence, and exposed to lipopolysaccharide (LPS) 0.5 or 1 *μ*M; to synchronize the circadian clock, cells were then treated with 1 *μ*M dexamethasone (dex, Sigma-Aldrich, Hamburg, Germany) for 1 hour.

Ozone treatment was performed in previously LPS-treated-synchronized and control cells 18 hours after dex treatment in accordance with our previous work [12].

### 2.2. Ozone Exposure

O_3_ was generated from O_2_ by electrical corona arc discharge (ECO_3_ model CUV-01, Torino, Italy), as previously described [27]. The O_2_–O_3_ mixture (95% O_2_, 5% O_3_) was combined with ambient air and allowed to flow into a Teflon-lined exposure chamber, with the O_3_ concentration in chamber adjusted to the ppm needed for the experiment and continuously monitored by an O_3_ detector. Temperature and humidity were monitored during exposures (37°C and 45–55%, respectively).

### 2.3. Total Protein Extraction

Cells were seeded in 60 mm petri (1.5 × 10^6^ cells). After treatments, cells were detached and washed twice with ice-cold PBS 1x and total cell lysates were extracted in ice-cold solubilization buffer containing 20 mM Tris pH 8, 150 mM NaCl, 1% Triton X-100, 1 mM sodium orthovanadate, 1 *μ*g/ml leupeptin, 1 *μ*g/ml aprotinin, 1 *μ*g/ml pepstatin, 10 *μ*g/ml phenylmethylsulfonyl fluoride (PMSF), and 5 mM *β*-glycerophosphate (Sigma, Milan, Italy). After centrifugation (15 000 × g, 15 minutes at 4°C), the supernatants were collected. Protein concentrations were determined using the Bio-Rad protein assay kit (Bio-Rad, Milan, Italy).

### 2.4. Western Blot Analysis

After protein quantification, 40 *μ*g boiled proteins were loaded into 10% sodium dodecyl sulphate-polycrylamide electrophoresis gels and separated by molecular size. Gels were electroblotted onto nitrocellulose membranes and then blocked for 90 minutes in Tris-buffered saline, pH 7.5, containing 0.5% Tween 20 and 5% (*w*/*v*) skim milk powder. Membranes were incubated overnight at 4°C with the appropriate primary antibodies: NRF2 diluted 1 : 1000 (ABE413, Millipore, Billerica, Massachusetts) and NF-*κ*B-p65 diluted 1 : 1000 (sc372 Santa Cruz). The membranes were finally incubated with the peroxidase-conjugated secondary anti-Rabbit antibody (1 : 5000) for 1 hour. The bound antibodies were detected by chemiluminescence (Bio-Rad, Milan, Italy). *β*-Actin was used as loading control. Images of the bands were digitized using an Epson Stylus SX405 scanner, and the densitometry analysis was performed using ImageJ software.

### 2.5. Immunocytochemistry

Human keratinocytes were grown on coverslips at a density of 1 × 10^5^ cells/ml and after treatment fixed in 4% paraformaldehyde for 30 min at room temperature as previously described [28]. Cells were permeabilized for 5 min at RT with PBS containing 0.2% Triton X-100, blocked with 1% BSA in PBS at RT for 1 h, and then incubated with primary antibody (NF-*κ*B-p65, 1 : 100, sc372 Santa Cruz; NRF2 1 : 200 ABE413 Millipore) in PBS containing 0.5% BSA at 4°C overnight. After washing, coverslips were incubated with appropriate secondary antibody for 1 h at RT. Nuclei were stained with 1 *μ*g/ml DAPI (4′,6-diamidine-2′-phenylindole dihydrochloride, Sigma-Aldrich, Italy) for 1 min. Coverslips were mounted onto glass slides using antifade mounting medium 1,4 diazabicyclooctane (DABCO) in glycerin. Negative controls for the experiments were performed by omitting primary antibodies. Images were acquired and analyzed with Leica AF CTR6500HS (Microsystems) and analyzed with CellProfiler software.

### 2.6. NRF2 DNA-Binding Activity

NRF2 to DNA ARE were evaluated using the “TransAM Nrf2” ELISA kit (Active Motif, USA). NRF2 protein presented in cellular extract was incubated with oligonucleotides containing ARE sequencing, immobilized on a 96-well plate. A secondary antibody conjugated with a horseradish peroxidase provides a colorimetric output spectrophotometrically detected at 450 nm.

### 2.7. RNA Extraction and Gene Expression Analysis

Total RNA was isolated from confluent cells using TRIzol Reagent (Invitrogen), according to the manufacturer's protocol. The amount and quality of isolated RNA were analyzed by BioSpec-nano (Shimadzu, Kyoto, Japan). One microgram of DNase-treated RNA was used to perform cDNA synthesis, using the iScript cDNA Synthesis Kit (Bio-Rad, Milan, Italy). First-strand cDNA was PCR amplified with a CFX Real-Time PCR Detection System (Bio-Rad, Milan, Italy) using SsoFast EvaGreen Supermix (Bio-Rad Laboratories, Hercules, CA, USA). After amplification, a melting curve analysis to confirm the specificity of the amplicons was performed. We used the gene-specific primers for human *Ho1*, *Nqo1*, *Il1β*, *I11α*, *Il6*, *Il8*, and *18* s (Table 1).

The relative levels of each sample were calculated by the 2^–*ΔΔ*CT^ method (where CT is the cycle number at which the signal reaches the threshold of detection) [29]. Each CT value used for these calculations is the mean of three replicates of the same reaction.

### 2.8. Statistical Analysis

All the results were expressed as means ± SEM. Treatments, sampling time, and their interaction were tested by parametric (one-way ANOVA and unpaired *t*-test) and nonparametric (Mann-Whitney *U*-test) tests. Normality was tested by the Kolmogorov–Smirnov test (*P* < 0.05). Tukey's Multiple Comparison Test was applied as the post hoc test. *P* values < 0.05 were considered statistically significant. Data were analyzed using the software GraphPad Prism 4.0 (GraphPad Software, Inc., La Jolla, CA).

## 3. Results and Discussion

On the basis of our previous results [12] showing the association between circadian system and oxidative stress, we explored the crosstalk between NRF2 and NF-*κ*B in clock-synchronized and arrhythmic human keratinocytes exposed to O_3_.

**Figure 1:**
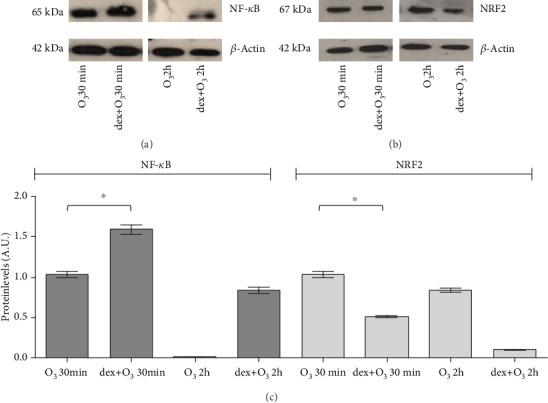
Expression of NRF2 and NF-*κ*B proteins in HaCaT cells exposed to ozone or dex+O_3_. Samples were harvested at two different time points (30 minutes and 2 hours after the treatments), and the protein expression was measured by Western blot. Representative Western blot of 3 different experiments is depicted in (a) and (b). Quantification of the NRF2 and NF-*κ*B bands is shown in (c). Data are expressed as mean ± SEM from three independent experiments. *β*-Actin was used as a loading control. ^∗^*P* < 0.05.

By Western blot analysis, as shown in Figure 1, we observed a significant increase in NF-*κ*B levels (+33%, *P* < 0.014, unpaired *t*-test) right after 30 min of O_3_ exposure in synchronized cells with respect to the arrhythmic keratinocytes. On note, at 2 hr time point, this effect was completely abolished in unsynchronized cells while was still evident in the synchronized ones. This result indicated that the response to O_3_ in synchronized cells is more long lasting, as suggested by the higher level of total NF-*κ*B. This data further indicated the possibility that the cells are more prone to reactivate NF-*κ*B (nuclear translocation) in the eventuality of a further challenge. Indeed at this time, it is not possible to discriminate between NF-*κ*B nuclear and cytoplasmic levels.

As the literature strongly indicate the crosstalk between NF-*κ*B and NRF2, we wanted to also determine the effect of O_3_ and the synchronization on its levels. As depicted in Figure 1(b), NRF2 levels were significantly increased by O_3_ exposure at earlier time point (30 min) in dex-treated cells with respect to the keratinocytes unsynchronized (+47% *P* < 0.0085, unpaired *t*-test). Of note, this trend was also noticed at later time point up to 2 hr. These results suggest that clock synchronization provides a more prompt ability of the cells to increase NRF2 confirming what has been already shown in previous work by Benedusi et al. [12].

**Figure 2:**
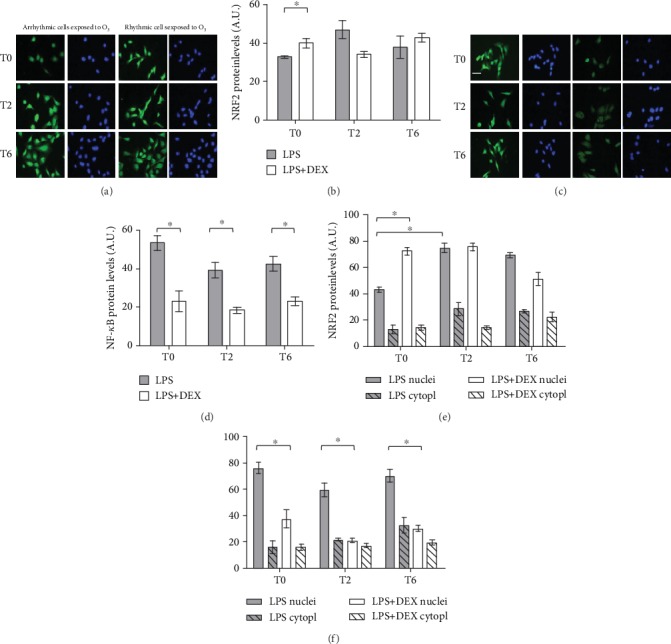
Immunofluorescence for NRF2 and NF-*κ*B in dex-synchronized HaCaT cells and arrhythmic ones chronically inflamed with LPS 1 *μ*g/ml and exposed to O_3_. Representative pictures of three different experiments are presented. Nuclei (blue) were stained with DAPI. Magnification 40x, scale bar = 50 *μ*m. Samples were harvested at different time points (0, 2, and 6 hours after ozone exposure). Quantification of fluorescence in total HaCaT cells (b, d), cytoplasm, and nucleus fractions (e, f) of NRF2 and NF-*κ*B proteins is shown. Data are expressed as mean ± SEM from three independent experiments. ^∗^*P* < 0.05.

Comparing the NF-*κ*B and NRF2 plots, it is easy to observe an opposite trend among the two transcription factors that confirms their interdependency. At this stage, we did not evaluate the nuclear levels of NF-*κ*B and NRF2 because the intent of this study is to understand the role that an inflammatory stimulus (LPS treatment) has on circadian clock modulation of antioxidant pathway in response to O_3_ insult. Indeed, this issue is shown and discussed below (Figure 2).

To test the appropriate doses of LPS to be used in our experimental protocol, keratinocytes were treated with 0.5 and 1 *μ*M of LPS for 24 hours and cytotoxicity was then evaluated by lactate dehydrogenase (LDH) release assay, in synchronized and arrhythmic cells. No significant cytotoxicity was observed at 0.5 and 1 *μ*M of LPS (data not shown). These data prompted us to use 1 *μ*M as a dose of LPS treatment to induce chronical inflammation without irreversibly damage the cells.

To better elucidate the interplay mechanisms between circadian clock, NRF2, and NF-*κ*B pathways, we induced chronic inflammation in keratinocytes by LPS treatment, and we then analyzed the inflammatory and antioxidant pathways upon acute O_3_ exposure in clock-synchronized and clock-desynchronized cells.

Since dex used for cell clock synchronization is a steroidal anti-inflammatory drug, we first confirmed that 1-hour dex-treatment on keratinocytes does not significantly alter the inflammatory pathway in terms of NF-*κ*B activation (data not shown). This result is in accordance with previous study [30] where it has been demonstrated that exposure of cutaneous cells to dex induced the transient translocation of NF-*κ*B from the cytoplasm to the nucleus. These changes were transitory, as in cells treated with dex for 1 h, the protein rapidly returned to its cytoplasmic location.

It is well documented that O_3_ insult in keratinocytes induces cellular defensive mechanisms by the activation of NRF2 [31–33]. Moreover, our previous results demonstrated that in clock entrained cells, the level of NRF2 was significantly lower than in arrhythmic keratinocytes after exposure to 0.2 ppm O_3_ [12], suggesting a less efficient antioxidant response compared to the entrained cells. Since we observed a specular trend in the NF-*κ*B expression level, we analyzed the crosstalk of the three pathways in entrained and arrhythmic chronically inflamed HaCaT cells after O_3_ insult.

By immunocytochemistry (Figures 2(a) and 2(b)), we observed an evident increase in the total NRF2 protein level in entrained cells (*P* = 0.0226; unpaired *t*-test) immediately after O_3_ exposure (T0). Conversely, a lower expression of total NF-*κ*B (Figures 2(c) and 2(d)) at all time points was detected in inflamed-synchronized cells, compared to arrhythmic ones (*P* < 0.003; unpaired *t*-test).

As both NRF2 and NF-*κ*B mediate the transcriptional response of cells once activated (nuclear levels) [34], we next quantified their subcellular localization after O_3_ exposure in inflamed-synchronized and desynchronized cells. As expected, nuclear protein levels of both NRF2 and NF-*κ*B raised up immediately after the insult compared to cytoplasm compartment; however, immunofluorescence staining revealed that (Figure 2(e)) NRF2 protein was significantly higher in the nuclear compartment in rhythmic cells with respect to arrhythmic ones (+35% T0; *P* < 0.007, Mann-Whitney *U*-test). On the other hand, in arrhythmic cells, NRF2 nuclear translocation reached its maximum level 2 hours later, suggesting a slower and less efficient response (+37% from T0 to T2; *P* < 0.008, Mann-Whitney *U*-test). Moreover, in desynchronized cells, we observed a NF-*κ*B higher nuclear level right after O_3_ insult, when compared to entrained cells (+42%; *P* < 0.01, Mann-Whitney *U*-test). This intracellular localization trend of NF-*κ*B was maintained at T2 and T6 (*P* < 0.01, Mann-Whitney *U*-test). Taken together, these results underpin the mutual interplay of these two pathways under oxinflammatory conditions.

It is well established that the cellular actions of NRF2 and NF-*κ*B signaling pathways are in opposition [35–37], with reciprocal inhibition mechanistic relationship, but the principal modulator that plays a pivotal role is yet to be defined. Mimicking a scenario of chronic inflammation and circadian deregulation, we speculated that keratinocytes exposed to acute oxidative stress displayed slower defensive response in terms of nuclear translocation of NRF2. Within the nucleus, NRF2 exerts its transcriptional function by binding to the antioxidant response element (ARE 5′-TGACXXXGC-3′) in the promoter region of NRF2 antioxidant target genes (such as NAD(P)H:quinone oxidoreductase1, NQO1, or heme oxygenase 1, HO1) [38].

**Figure 3:**
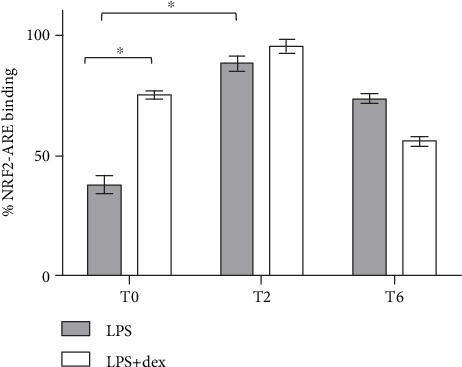
NRF2 binding to DNA antioxidant response element (ARE) in dex-synchronized HaCaT cells and arrhythmic ones chronically inflamed with LPS 1 *μ*g/ml and exposed to O_3_. Samples were harvested at different time points (0, 2, and 6 hours after ozone exposure). Data are presented as mean ± SEM of three independent experiments in triplicate. ^∗^*P* < 0.05.

A TransAM ELISA was performed to endorse the DNA-binding activity of NRF2 upon O_3_ insult. As shown in Figure 3, immediately after O_3_ exposure, the DNA-binding activity of NRF2 was higher in synchronized cells with respect to arrhythmic keratinocytes (+37% vs. arrhythmic cells; *P* = 0.001, unpaired *t*-test). Differently, in desynchronized cells, DNA-binding activity increased at T2 (+50% vs. T0; *P* < 0.001, unpaired *t*-test). At T6, in entrained cells, NRF2 DNA-binding activity declined much faster than in arrhythmic cells, indicating a more efficient antioxidant response in chronically inflamed dex-synchronized human keratinocytes.

**Figure 4:**
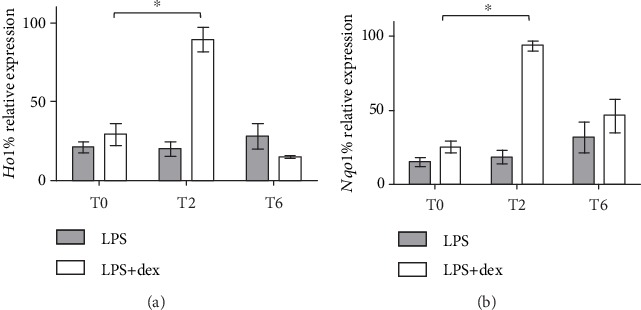
Relative expression (in percentage) of *Ho1* (a) and *Nqo1* (b) mRNA in dex-synchronized HaCaT cells and arrhythmic ones chronically inflamed with LPS 1 *μ*g/ml and exposed to O_3_ was determined using qRT-PCR. Samples were harvested at different time points (0, 2, and 6 hours after ozone exposure). For all genes, the constitutively expressed 18S rRNA was used to normalization. For each time point, the mean ± SEM of three independent experiments is shown. ^∗^*P* < 0.05.

In this light, as displayed in Figure 4, we explored the expression of NRF2-depending phase II enzymes after O_3_ exposure in chronically inflamed cells by analyzing the levels of *Ho-1* and *Nqo1* in entrained and arrhythmic conditions.

qRT-PCR analysis of phase II enzyme expression revealed that both genes were strongly induced 2 hours after O_3_ insult (+60% for *Ho1* and +70% for *Nqo1*; *P* < 0.005 T2 vs. T0, unpaired *t*-test) in synchronized keratinocytes. By contrast, neither *Ho1* nor *Npqo1* gene expression resulted a significant effect after O_3_ exposure in arrhythmic cells. These results are in agreement with our previous observation of NRF2 nuclear translocation and DNA-binding activity immediately after O_3_ insult in rhythmic cells.

It is well known that *Ho1* have significant anti-inflammatory effects mediated by NRF2 as it has been demonstrated in mouse myoblasts exposed to H_2_O_2_ [39] and in mouse peritoneal macrophages treated with LPS [40]. Starting from this knowledge, we analyzed the expression of genes involved in the inflammatory response induced by the activation of NF-*κ*B.

**Figure 5:**
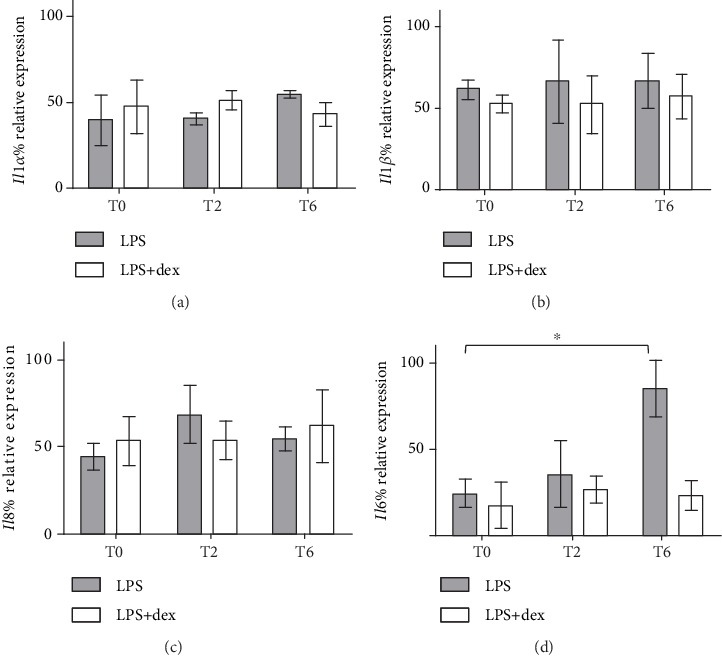
Relative expression (in percentage) of *Il1α* (a), *Il1β* (b), *Il8* (c), and *Il6* (d) mRNA in dex-synchronized HaCaT cells and arrhythmic ones chronically inflamed with LPS 1 *μ*g/ml and exposed to O_3_ was determined using qRT-PCR. Samples were harvested at different time points (0, 2, and 6 hours after ozone exposure). For all genes, the constitutively expressed 18S rRNA was used to normalization. For each time point, the mean ± SEM of three independent experiments is shown. ^∗^*P* < 0.05.

As shown in Figure 5, *Il1β*, *Il8*, and *Il1α* mRNA levels did not change over the different time points neither in rhythmic nor in arrhythmic cells. Intriguingly, *Il6* expression profile increases significantly from T0 to T6 in chronically inflamed asynchronous cells exposed to O_3_ (*P* = 0.0061; one-way ANOVA), while no differences were observed in entrained cells.

Recent work indicates that circadian and immune functions are highly interconnected [41]. However, the consequences of a disrupted circadian environment for proper immune functions remain unclear. Moreover, IL-6 is a key signal that mediates mutual feedback interactions between inflammation and modulation of peripheral circadian clocks [42]. Our data falls into this picture suggesting that keratinocytes exhibit LPS-induced IL-6 release after oxidative challenge, when the circadian rhythm is disrupted.

**Figure 6:**
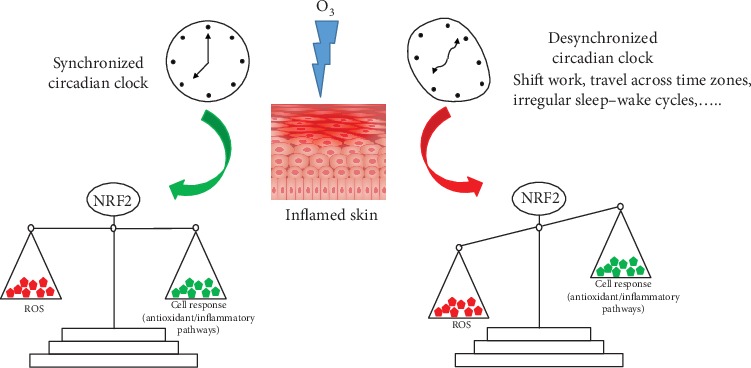
Schematic summary of the discussion and conclusion sections. We suggest the possible role of the circadian clock in O_3_-induced skin inflammation. In particular, we propose that a synchronized circadian clock facilitates the protective role of NRF2 in terms of a more efficient antioxidant response against environmental insult and of a regulation of the cellular damage resulting from chronic inflammation.

In summary (Figure 6), in this experimental scenario, we suggest that a synchronized circadian clock not only facilitates the protective role of NRF2 in terms of a faster and more efficient antioxidant response against environmental insult but also moderates the cellular damage resulting from a condition of chronic inflammation.

## 4. Conclusions

The general involvement of aberrant circadian clock has been linked to the development or at least the progression of several pathologies [8, 9]. Preliminary studies have suggested the role of clock synchronization in protecting the skin from exogenous source damage [12]. In particularly, several proinflammatory cutaneous conditions (such as psoriasis, eczema, cutaneous rushes, and atopic dermatitis) have been attributed to outdoor stressor exposure such as O_3_ [43]. The role of O_3_ exposure either in developing an inflammatory process or in progressing an inflammation already present is still under investigation although its connection with proinflammatory mediators has been well demonstrated [27]. The present study intends to suggest the possible role of the circadian clock in O_3_-induced skin inflammation. It is also possible that those results can be extrapolated to other pollutants such as cigarette smoke and particulate matters, as they have been demonstrated to have similar mechanisms of action at the cutaneous levels [44].

## Figures and Tables

**Table 1:** 

*Ho1*	Forward: TTGCTTTGGCGAGCTCTTTT Reverse: TCTGATGCCAAACACCCCA

*Nqo1*	Forward: CTGATCGTACTGGCTCACTC Reverse: AACAGACTCGGCAGGATAC

*Il1β*	Forward: ACA GAT GAA GTG CTC CTT CCA Reverse: GTC GGA GAT TCG TAG CTG GAT

*Il1α*	Forward: GGAGCTTGTCACCCCAAACT Reverse: TCCGAAGTCAAGGGGCTAGA

*Il6*	Forward: GTAGCCGCCCCACACAGA Reverse: TCTGAGGTGCCCATGCTAC

*Il8*	Forward: GGTGCAGTTTTGCCAAGGAG Reverse: TTCCTTGGGGTCCAGACAGA
*18S*	Forward: CGAGCCGCCTGGATACC Reverse: CATGGCCTCAGTTCCGAAAA

## Data Availability

the data used to support the findings of this study are available from the corresponding authors upon request.
